# Occupational Hazards Education for Nursing Staff through Web-Based Learning

**DOI:** 10.3390/ijerph111213035

**Published:** 2014-12-12

**Authors:** Chen-Yin Tung, Chia-Chen Chang, Jin-Lain Ming, Keh-Ping Chao

**Affiliations:** 1 Department of Health Promotion and Health Education, College of Education, National Taiwan Normal University, Taipei 10610, Taiwan; E-Mails: s09144@ntnu.edu.tw (C.-Y.T.); babalabear@gmail.com (C.-C.C.); 2 Nursing Department, Taipei Veterans General Hospital, Taipei 11217, Taiwan; E-Mail: jlming@vhgtpe.gov.tw; 3 Department of Occupational Safety and Health, College of Public Health, China Medical University, 91 Hsueh-Shih Rd., Taichung 40402, Taiwan

**Keywords:** online education, occupational hazard, KAP, hospital

## Abstract

This study aims to explore the efficiency of using online education as an intervention measure to prevent occupational hazards in a clinical nursing setting. The subjects were 320 female nursing staff from two hospitals in Taiwan. The questionnaire results indicated that the subjects primarily experienced human factor occupational hazards, as well as psychological and social hazards. Specifically, 73.1% and 69.8% of the subjects suffered from poor sleep quality and low back pain, respectively. After web-based learning, the experimental group had higher post-test scores than the control group in terms of knowledge, attitudes, and practices (KAP). However, there was only a significant difference (*p* < 0.05) in their knowledge about the prevention of occupational hazards. It is suggested that an online discussion may enhance nursing staff’s participation in web-based learning, and further facilitate their comments on negative factors. The findings can highly promote nursing staff’s attitudes and practices toward preventing occupational hazards through web-based learning.

## 1. Introduction

As the workplace for medical treatment and health improvement, hospitals have high standards for safety and hygiene. However, healthcare workers are confronted by numerous occupational hazards due to the unique nature of their work [1]. The convenience of accessing medical information may cause the healthcare workers to neglect the health risks in their workplaces. According to a national survey on occupational hazards in the United States, the incidence rate of occupational injury and illness for the medical and healthcare industry was as high as 6.6% and ranked forth out of 56 service industries in 2012 [2]. Moreover, compared with workers in other industries, healthcare workers reported more incidents of back strain, dermatitis, infectious hepatitis, infectious diseases, psychological disorders, eye diseases, flu, and toxic hepatitis.

Online learning has been widely applied in the professional training and continuing education for healthcare workers. The benefits of online learning for nursing staff include: (1) the provision of a convenient learning environment that is free from the restrictions of time and space, which is useful for nursing staff who have difficulty attending regular classes; (2) most nursing staff have intermediate to advanced level computer literacy, so that they can easily access online information; and (3) hospitals have well-equipped computers and Internet connections, thus facilitating the promotion of a web-based learning program [3,4,5]. Chuang and Tsao [3] indicated that online learning by nursing staff could efficiently shorten their learning time, lead to a heightened feeling of achievement, and improve knowledge and skills, thus resulting in a high satisfaction with their courses. Raes *et al.* [4] found that the efficacy of students’ voluntary online learning is equivalent to the conventional classroom teaching. Therefore, it is feasible to develop an online learning program for nursing staff.

According to the Global Information Technology Report, Taiwan is among the top ten countries with complete Internet resources, which include the Internet environment, readiness, usage, and impact [6]. On average, seven out of ten people in Taiwan have Internet-usage experience, and the majority of Internet users have a college degree or higher [7]. For that reason, the intervention of online education is appropriate for the healthcare workers in Taiwan.

In this study, a quasi-experimental design was conducted among 320 female nursing staff at two hospitals in Taipei City, Taiwan. The subjects were divided into an experimental group and a control group. Theories on health behaviors that may influence learners’ knowledge, attitudes and practices (KAP) were used to design the teaching materials on occupational safety and health for nursing staff’s needs. The experimental group received online education for the prevention of occupational hazards. The purposes of this study were to investigate the efficiency of online education as an intervention measure on preventing occupational hazards, as well as to understand nursing staff’s experiences of occupational injuries. The findings can help to promote nursing staff’s safety and health in their workplaces.

## 2. Method

### 2.1. Research Subjects

This study adopted a pre/post-test control group design. The subjects were chosen among nursing staff from two hospitals in Taipei City. Each hospital has approximately 300 beds. One hospital was randomly assigned as the experimental group and the other one was the control group. This study was conducted in accordance with the Declaration of Helsinki, and was approved by the Ethical Committee of the National Taiwan Normal University (IRB approval number P970205). Informed written consent was provided by the subjects prior to their inclusion in this study.

Schumacher *et al.* [8] found that there were significant gender differences in Internet experiences and attitudes among college students in the United States. Only two and three male nurses worked in the experimental and control hospitals, respectively, so the male nursing staff was excluded from this study. In addition, nursing staff without good experience in using the Internet, *i.e*., five staff members, were excluded from the experimental group. Finally, a total of 320 full-time nursing staff, including 150 in the experimental group and 170 in the control group, were randomly selected by the administrators from the medical departments in proportion to the total number of nursing staff in each department.

The control group participated in a 4 h regular education program for occupational safety and health, which was undertaken as the conventional classroom teaching and used the teaching materials developed for web-based learning. Through the website, the experimental group received Internet education for the prevention of occupational hazards over a 6-week period. Head nurses would remind the nursing staff in the experimental group in person or through e-mail to visit the online-learning website.

### 2.2. Theoretical Framework

As shown in Table 1, the content of Internet education for preventing occupational hazards was based on the health behavior theories, such as the health belief model, the persuasive communication model, the social cognitive theory, the social support theory, and the social marketing theory. The health belief model, incorporating the self-efficacy theory, underpinned the theoretical framework of this study. The health belief model suggests that people’s beliefs about health problems, perceived benefits of action, and barriers to action explain the engagement in health-promoting behavior [9]. Therefore, understanding and clarifying the positive and negative factors could enhance the nursing staff’s knowledge, benefits, and hindrances of preventing occupational hazards. According to the persuasive communication model, Internet-based learning can increase the nursing staff’s awareness of occupational hazards by enhancing their motivation to visit the website. Additionally, the framework of this study was grounded in social cognitive theory and social support theory. Interventions based on these theories, such as setting goals for the prevention of occupational hazards, recording details of hazards, and sending e-mail to remind the participation, could promote the nursing staff’s attitudes and practices towards the prevention of occupational hazards.

**Table 1:** Theoretical framework of the website for the prevention of occupational hazards.

Educational Objective	Educational Theory	Intervention Variable
Enhance the subjects’ motivation to visit the website	Persuasive communication model	Awareness of occupational hazards
Set goals for the prevention of occupational hazards and record details of hazards	Social cognitive theory	Attitudes towards the prevention of occupational hazards
Understand relevant information concerning the prevention of occupational hazards	Health belief model	Knowledge of occupational hazards Benefits of preventing occupational hazards Hindrances to the prevention of occupational hazards
Clarify positive and negative factors for the prevention of occupational hazards	Health belief model	Benefits of preventing occupational hazards Hindrances to the prevention of occupational hazards
Use online games to simulate practices for the prevention of occupational hazards	Self-efficacy	Practices to prevent occupational hazards
Use successful examples to enhance colleagues’ confidence	Social support theory	Social support to the prevention of occupational hazards Practices to prevent occupational hazards
Use e-mail remainders to reinforce the participation	Social support theory	Attitudes towards preventing occupational hazards Social support for the prevention of occupational hazards
Give out complimentary gifts to attract subjects to visit the website	Social marketing theory	Attitudes towards the prevention of occupational hazards

The website content included descriptions of the types of occupational hazards, five online videos and numerous photos that demonstrated the practices to prevent occupational hazards, a blog to share experiences related to occupational hazards, five interactive games, and the website click rate chart. The online teaching materials were reviewed by seven experts, whose professional backgrounds included occupational health, nursing practice, and health education, to ensure that they met the educational objectives shown in Table 1. In addition, the website content was assessed by the head nurses in the experimental hospital. The comments from the experts and head nurses were used to modify the website content.

### 2.3. Pre- and Post-Test

The questionnaires were designed to collect information on individual characteristics (age, level of education), work conditions (workplace, duration of work at the current position, nature of shift work, daily workload, *etc.*), and perceived experiences of occupational hazards. In addition, the pre-test and post-test encompassed the subjects’ awareness, KAP, comments on positive factor, comments on negative factor, self-efficacy and social support toward the prevention of occupational hazards. The experimental and control groups used the same questionnaires for the pre- and post-test, which included 121 closed-ended questions. Respondents were asked to decide where they fit along a scale continuum based on the questions from different categories. The questions pertaining to knowledge and social support were answered with a simple “Yes” (1) or “No” (2). The questions pertaining to awareness, self-efficacy, attitudes, and positive/negative comments were rated by the subordinates on a 5-point scale ranging from “Strongly disagree” (1) to “Strongly agree” (5) based on the Likert scale. For the questions of practices, an ordered set of answers was rated on a 7-point scale ranging from “Never” (1) to “Always” (7). For example, the ordinal scale was 1–7 in the response to a question such as “I always wear protective gloves when working.” The questionnaire for the subjects’ experiences of occupational hazards had a multiple-choice format and was conducted along with the pre-test.

The reliability and validity of the questionnaire were confirmed. The degree of discrimination for each item on the knowledge toward the prevention of occupational hazards was between 0.19 and 0.67, while the degree of difficulty was between 0.35 and 0.91. The Cronbach’s coefficient alphas for other variables were between 0.7 and 0.95, indicating that the internal consistency reliability was high. The subjects completed the pre-test and the questionnaire pertaining to their experiences of occupational hazards one week prior to the commencement of the six-week Internet education program. The post-test was carried out one week after program completion.

### 2.4. Data Analysis

The data were analyzed using the Statistical Package for Social Sciences (SPSS) version 19.0. The descriptive statistics and inferential statistical methods were used according to the research purposes. Descriptive statistics indicated the frequency distribution and percentage of the nursing staff’s background and experiences of occupational hazards. The chi-square test and paired *t*-test were employed to examine the consistency in nominal variables and interval variables, respectively, for the experimental group and the control group. To perform analysis of covariance (ANCOVA), the pre-test score of each variable was designated as the covariance, and each group was the independent variable to understand the effects of educational intervention on the dependent variables of the subjects.

## 3. Results and Discussion

### 3.1. Background

Table 2 shows the description of individual characteristics of the subjects. The independent *t*-tests and chi-squared tests showed no significant differences between the experimental group and control group on the characteristics, except for the duration of work at the current position (*p* < 0.05). The average work duration of the nursing staff in the experimental group was 4.67 years, while that of those in the control group was 3.57 years. In addition, their experiences in nursing work ranged from 0.5 to 32.5 years, with an average of 7.8 and 6.34 years for the experimental group and control group, respectively. The common educational level was junior college or higher. The nursing staff in the experimental and control groups cared for 4 to 7 patients during an 8–9 h shift. Moreover, 87.4% of the subjects in the experimental group and 86% in the control group had to work on varying shifts. It was difficult for them to attend regular classes on occupational safety and health.

**Table 2:** Basic information of the subjects.

Item	Experimental Group	Control Group	*X^2^*	T
Age	29.04	27.93		2.08
Education level (%)			0.92	
Junior college	35.7	38.8		
Bachelor degree	61.1	60.0		
Master degree	3.2	1.2		
Performing administrative duty (%)			1.63	
Yes	5.6	6.7		
No	94.4	93.3		
Years of experience for nursing work	7.80	6.34		1.54
Years of experience at the current position	4.67	3.57		9.00 *
Workplace (%)			4.07	
General medical wards	41.6	35.9		
Intensive care units	10.4	9.5		
Operation rooms	11.5	15.5		
Others (delivery rooms, emergency rooms)	36.5	39.1		
Nature of shift work (%)			0.45	
Fixed day shift	10.5	12.8		
Fixed evening shift	2.1	1.2		
Fixed night shift	0.0	0.0		
A rotating shift schedule	87.4	86.0		
Daily workload (hour/day)	8.43	8.53		0.00
Days of monthly leaves (day/month)	8.00	8.10		0.55
Number of patients per working day (capita/day)	5.10	4.80		0.69

Note: * *p* < 0.05.

### 3.2. Experiences of Occupational Hazards

Table 3 shows the subjects’ experiences of occupational hazards. The frequency distribution of their experiences of occupational hazards was in a descending order of: human factor hazards > psychological and social hazards > chemical hazards > infectious and biological hazards > physical hazards. Several researchers indicated that the main occupational risks for healthcare workers have changed from chemical, physical, and biological hazards to human factor, as well as psychological and social hazards [10,11]. As shown in Table 3, 69.8% of the nursing staff had experiences of low back pain. Feng *et al.* [12] found that 66% of nursing staff in Taiwan were troubled by low back pain due to poor posture and repetitive activities at work over a long time. In addition, Table 3 shows that 60.4% and 61% of the subjects had experiences of varicose veins and muscle strains, respectively.

In terms of psychological and social hazards, over 60% of the subjects reported being in a bad mood (65.9%), feeling anxious (65.4%), and having reduced social activities (65.4%). Because over 86% of the subjects had to work on shifts, it is plausible that 73.1% of the nursing staff reported being troubled by poor sleep quality. As shown in Table 3, 22% of the subjects had the experience of medical disputes. During the past decades, the number of cases involving medical disputes has increased dramatically in several countries, such as the UK, the USA, Australia, New Zealand, Japan and Taiwan [13].

**Table 3:** Research subjects’ experiences of occupational hazards.

Types of Hazards	N ^†^ (%)	Types of Hazards	N ^†^ (%)
Chemical hazards		Infectious and biological hazards	
Vertigo and nausea	78 (42.9)	Upper respiratory tract infection	74 (40.7)
Irritated eyes	68 (37.4)	Scabies	17 (9.3)
Respiratory allergy	59 (32.4)	Fungal infection	9 (4.9)
Throat discomfort	57 (31.3)	Infectious hepatitis	9 (4.9)
Occupational dermatitis	35 (19.2)	Tuberculosis	4 (2.2)
Kidney and liver Discomfort	11 (6.0)	Physical hazards	
Nervous system damage	8 (4.4)	Burns and scalds	24 (13.2)
Malicious tumour	1 (0.5)	Skin redness	22 (12.1)
Psychological and social hazards		Reduced white blood cell count	12 (6.6)
Poor sleep quality	133 (73.1)	Malicious tumor	2 (1.1)
Bad mood	120 (65.9)	Human factor hazards	
Anxiety	119 (65.4)	Low back pain	127 (69.8)
Reduced social activities	119 (65.4)	Muscle strain	111 (61.0)
Medical dispute	40 (22.0)	Varicose veins	110 (60.4)
Physical violence	31 (17.0)	Eye fatigue and harm to eyesight	99 (54.4)
Sexual harassment	31 (17.0)	Falling on the floor	59 (32.4)

Note: ^**†**:^ Number of subjects.

Medical disputes can result in work-related stress for nursing staff. If work stress is not dealt with properly, it could lead to mental exhaustion and physical effects. According to the studies of questionnaires, Chen *et al.* [11] found that the prevalence rates of physical violence varied from 5.3% to 21% in England, Hong Kong and China. Table 3 indicates that 17% of the subjects reported having experienced physical violence and sexual harassment in the workplace. Even though the frequency distribution of subjects’ experiences of physical violence was not significantly high in comparison with other occupational hazards, it is suggested that education in occupational hazards prevention should focus on reducing workplace violence.

### 3.3. Participation in Internet Education

The education website designed by this study could record the number of visits by each subject. Although the head nurses weekly reminded the nursing staff in the experimental group, the records indicated only 115 (76.7%) of the 150 subjects visited the website with an average of 2.65 times. Among all articles on the website, the articles related to workaholism were most visited, *i.e*., 46 times. Finally, a total of 91 (60.7%) subjects in the experimental group completed the web-based learning program. In a survey on distance education, Tao and Yeh [14] found that approximately 20%–50% of learners discontinued their learning during the course, for reasons such as lack of time for learning, lack of motivation to learn, having no problem-solving skills, having no peer support, or a poor curriculum design. Kirkley and Stein [15] indicated that the fear of having new job duties added to their heavy clinical workloads is a possible reason behind nursing staff’s reluctance to participate in computer and web-based activities. Cheng *et al.* [16] pointed out that a learner without reasonable motivation is quite difficult to learn online actively. Therefore, there is a need to develop an organizational learning environment, such as policies and group enrollment, to increase the nursing staff’s motivation to use web-based learning.

### 3.4. Effects of Online Education Intervention

Table 4 shows that the post-test scores of the experimental group were higher than those of the control group. By controlling the pre-test scores, it was found that the post-test scores of the experimental and control groups were significantly different (*p* < 0.05) in their knowledge, awareness, comments on positive factors, self-efficacy and social supports toward the prevention of occupational hazards. After receiving Internet education, the experimental group, however, did not show significantly higher scores of attitudes, practices, and comments on negative factors than the control group. According to the technology acceptance model (TAM), new technologies can enhance users’ perceived ease-of-use and perceived usefulness for a system [17,18]. Therefore, the nursing staff in the experimental group received novel Internet education, and their knowledge score was significantly higher (*p* < 0.001) than that of the control group.

**Table 4:** Research subjects’ post-test on prevention of occupational hazards.

Item	Post-Test Mean		Adjusted Mean		ANCOVA F Ratio
	Experimental Group	Control Group	Experimental Group	Control Group	
Awareness	124.89	117.62	125.68	116.78	6.50 *
Knowledge	7.66	6.74	7.62	6.80	9.86 **
Attitudes	28.78	28.13	28.78	28.13	1.22
Practices	45.06	43.47	44.70	43.86	1.98
Comments on positive factor	37.04	35.40	36.97	35.49	4.58 *
Comments on negative factor	30.76	30.56	30.95	30.34	0.84
Self-efficacy	21.51	19.96	21.36	20.14	4.92 *
Social support	5.21	4.52	5.15	4.59	6.19 *

Notes: * *p* < 0.05; ** *p* < 0.001.

Table 5 compares the post-test scores of the subjects’ awareness toward the prevention of chemical, physical, infectious, biological, psychological and social hazards, as well as human factors. The experimental group had better performance on the hazard awareness than the control group did. However, the experimental group and the control group showed a significantly statistical difference (*p* < 0.05) only on their awareness toward the infectious and biological hazards. The online videos and photos showed the practical prevention of the needle stick injury. Additionally, there were step-by-step demonstrations of how to use the respiratory protective equipment in the workplace. The nursing staff of the experimental group could repeat web-based learning of teaching videos. Liu [19] found that teaching videos of new issues which feature converted professional knowledge can enhance the learner’s performances. Therefore, the visual stimuli of Internet education could help nursing staff in the experimental group to more easily acquire the information on preventing infectious and biological hazards. This can be the reason that the post-test score of the experimental group was significantly higher than that of the control group on the awareness toward infectious and biological hazards.

**Table 5:** Analysis of research subjects’ awareness toward prevention of occupational hazards.

Types of Hazards	Post-Test Mean		Adjusted Mean		ANCOVA F Ratio
	Experimental Group	Control Group	Experimental Group	Control Group	
Biological hazards	26.02	24.42	26.08	24.36	4.08 *
Chemical hazards	28.95	27.53	29.07	27.40	2.63
Physical hazards	20.80	19.61	20.69	19.72	1.33
Human factors hazards	21.54	20.96	21.52	20.98	0.93
Psychological & social hazards	26.89	25.93	27.08	25.73	0.11

Note: * *p* < 0.05.

For the experimental group, the nursing staff received weekly reminders from the head nurses, and shared their experiences of occupational hazards on the website’s blog, giving them an opportunity to interact with each other. In addition, the nursing staff could feel the concerns of their colleagues and the hospital on their health. This may result in an effect on their post-test scores on social support and comments on positive factors toward the prevention of occupational hazards. Bandura [20] developed the social cognitive theory and indicated that social support is vital to self-efficacy. Social support from the colleagues and hospital of the experimental group could be positively related to nursing staff’s self-efficacy. Therefore, it is plausible that self-efficacy of the post-test for the experimental group was significantly higher than the control group (*p* < 0.05).

### 3.5. Attitudes and Practices of Subjects

As shown in Table 4, the post-test scores of the experimental group on attitudes, practices, and comments on negative factors were higher than those of the control group. However, the difference between these two groups did not show statistical significance. According to the social cognitive theory, Bandura [20] indicated that social support influences behavior through self-efficacy. In addition, Shih [21] found that self-efficacy indirectly affected individuals' behavioral intentions and attitudes toward web-based learning. For the experimental group, the scores of self-efficacy and social support in the post-test were significantly higher than those of the control group (*p* < 0.05). It is speculated that comments on negative factors may play an influential role in nurses’ attitudes and practices toward web-based learning for occupational education.

Several studies have found that the discussion through online teaching is a critical dimension of web-based learning to enhance the learners’ achievement and satisfaction [22,23,24]. Cook *et al.* [24] indicated that the most effective e-learning methods for health professionals are those that include interactivity and feedback. In this study, the blog, created for the experimental subjects to share their experiences of occupational hazards, lacked an online discussion which could provide solutions to the problems that they encountered during the web-based learning program. Therefore, the post-test score of the experimental group was not significantly higher than that of the control group in the comments on negative factors. A lack of an online discussion on the website might further result in a low completion rate (60.7%) of online courses for the nursing staff in the experimental group.

## 4. Conclusions

Among the 320 female nursing staff participated in this study, most of them experienced occupational hazards due to human factor as well as psychological and social hazards. Moreover, 73.1% and 69.8% of them had the experiences of poor sleep quality and lower back pain, respectively. After completing the web-based learning program, the subjects of the experimental group had higher post-test scores than those of the control group. However, the experimental group and the control group did not show significant differences in their attitudes and practices toward the prevention of occupational hazards. A possible explanation for this result may be that the education website lacked an online discussion forum which could offer solutions to the problems encountered by the nursing staff in the prevention of occupational hazards. In addition, the lack of an online discussion may result in a low completion rate of the web-based learning program. Hence, setting up an online discussion forum for the education website may facilitate nursing staff’s comments on negative factors, which can raise their motivation to complete the learning program. This effort will stimulate nursing staff’s attitudes and practices toward the prevention of occupational hazards in hospitals.
